# Transforming urban planning processes and outcomes through creative methods

**DOI:** 10.1007/s13280-020-01436-3

**Published:** 2021-02-14

**Authors:** Steve Cinderby, Annemarieke de Bruin, Howard Cambridge, Cassilde Muhoza, Amanda Ngabirano

**Affiliations:** 1 Stockholm Environment Institute, Department of Environment and Geography, University of York, York, UK; 2 Stockholm Environment Institute, Head Office, World Agroforestry Centre, United Nations Avenue, Gigiri, P.O. Box 30677, Nairobi, 00100 Kenya; 3 The College of Engineering, Design, Art and Technology, Makerere University, University Rd, Kampala, Uganda

**Keywords:** Creative methods, Engagement, Inclusion, Mobility, Sustainable development goals, Urban planning

## Abstract

Inclusively delivering the sustainable development goals (SDGs) remains challenging, particularly in urban areas, where some of the most pressing concerns exist. To achieve the transformative SDG agenda, new methods are required to overcome current deficits in engagement around inclusion and equitable outcomes. Evaluating against theories of governance and inclusion, we test a mixture of digital and physical creative methods abilities to deliver co-designed solutions that influence mobility and road safety planning outcomes in East African cities. Greater inclusion led to improved interactions of citizens with decision makers, and the identification of novel, practical solutions, delivering some elements of transformation. Risks include creative methods being used to co-opt communities to official agendas, and institutional planning norms needing to adapt to respond to a wider range of stakeholders. Overall, where risks are mitigated, we recommend that using Creative Methods could localise SDG delivery, ensuring more equitable and effective outcomes from infrastructure development investments.

**Supplementary Information:**

The online version of this article (10.1007/s13280-020-01436-3) contains supplementary material, which is available to authorized users.

## Introduction

In our increasingly urbanising world, cities are where critical sustainability successes are most likely to be achieved. This has led to a significant focus on cities for delivering the UN Sustainable Development Goals (SDGs) (Klopp and Petretta 2017; Castán Broto et al. 2019). Rapidly growing urban populations are putting unanticipated pressures on city infrastructures and their operation, undermining some of the purported benefits of urbanisation (Cohen 2006). Including a wider cross section of residents in identifying these complex urban challenges, and co-designing solutions to address them, could help shape more sustainable future city spaces. Without considering the needs of a diverse range of voices, city planners risk identifying sub-optimal solutions that benefit a minority. Worse, the majority will be forced to improvise, potentially sub-optimal alternatives, to compensate for their exclusion. Such informal improvisations are readily apparent in Low- and Middle-Income Country (LMIC) where city growth is occurring most rapidly. Greater inclusion sits within the ambition of localising SDG implementation to specific contexts, places and communities (Klopp and Petretta 2017). SDG delivery would benefit from novel approaches, including Creative Methods (CMs), since urban planners have favoured engagement methods that are recognised for significant biases in participants and participation opportunities (Bobbio 2019), for example, public meetings, focus groups and exhibitions.

CMs are defined here as research that uses artistic modes of expression (using imagination to create objects, environments or experiences that can be shared with others) to explore ideas, represent possibilities and challenge current perspectives (Wang et al. 2017). This encourages transformative mindsets and frames discussions that encourage consideration of social change (Pearson et al. 2018). CMs’ value in allowing different stakeholders to comment upon complex issues (Hickey-Moody 2017) has been promoted by researchers for giving marginalised groups a voice, thereby helping to identify unconsidered or equitable solutions (Hammond et al. 2018). The diverse representation of stakeholders may help empower participants to challenge dominant knowledge, and open spaces for critical dialogue (Hammond et al. 2018). However, an improved evidence base, demonstrating CMs’ strengths and weaknesses, is required to justify investing development resources (Dunphy and Ware 2016; Daykin et al. 2017). This would potentially move these approaches from the status of ‘nice-to-haves’, towards an essential element of agencies’ toolkits for SDG delivery.

Our paper presents evidence from real-world experiments in East African urban settings, using mixed methods, to evaluate the performance of a suite of CMs and assess whether these under-valued approaches could deliver effective, implementable and transformative solutions. We interrogate our evidence to explore whether CMs could widen the range of participants involved in the urban planning process and deliver a transformation in inclusion. Secondly, we assess whether employing CMs could lead to significantly more equitable decisions on infrastructure development, resulting in a transformation in outcomes**.** Finally, we consider whether using CMs could overcome SDG delivery challenges, by achieving localised development gains for vulnerable communities in specific contexts and places.

## Theoretical framework

### Conceptualising the links between creative methods and transformations

Urban transformation has been defined as *“a process of fundamental irreversible changes in infrastructures, ecosystems, agency configurations, lifestyles, systems of service provision, urban innovation, institutions and governance”* (Elmqvist et al. 2019). Transformative change is required when failures (social, economic or environmental) in existing systems make new approaches essential (Pereira et al. 2018a). Transformation processes lead to either marked improvements in sustainability outcomes or fundamentally different forms of thinking, actions and systems incorporating greater equity (Fazey et al. 2018a; Pereira et al. 2018a). Transformations should ideally occur before limits to the adaptive capacity of existing systems are reached (Pelling et al. 2015). Shifting systems onto new trajectories requires collective, collaborative action across decision-making scales ranging from individuals’ mindsets and beliefs, through social norms and practices, to institutions and governance systems (Galafassi et al. 2018). Transformative change implies recognition of the multi-dimensional nature of sustainability challenges, whereby solutions must incorporate aspects of human and institutional behaviours, alongside infrastructure (Abson et al. 2017).

Methods that help frame problems, incorporate diverse knowledge, and equitably identify goals for change are critical needs in transformative processes (Abson et al. 2017). CMs activate rich thinking (Molderez and Ceulemans 2018) by creating liminal spaces where people are free to express themselves. This encourages experimentation, leading to new ideas (Lam et al. 2018; Pereira et al. 2018b). CMs facilitate the effective communication of concerns whilst also revealing community strengths or assets (Wang et al. 2000). We hypothesise that these purported potential benefits of CMs could contribute towards understanding current city problems holistically, leading to marked differences in outcomes (Fazey et al. 2018b) building transformative urban development capacity (Wolfram et al. 2016). Explicitly revealing urban failings could catalyse further use of CMs, by providing actionable knowledge to enable transformative change, leading to novel, context-specific solutions for overcoming local problems (Molderez and Ceulemans 2018). Critically, to achieve transformation at city-wide scales, we need to understand whether outputs from CMs can impact the views and actions of a range of relevant audiences, from general publics to policy and decision makers (Wang et al. 2000; Abson et al. 2017; Galafassi et al. 2018).

**Fig. 1:**
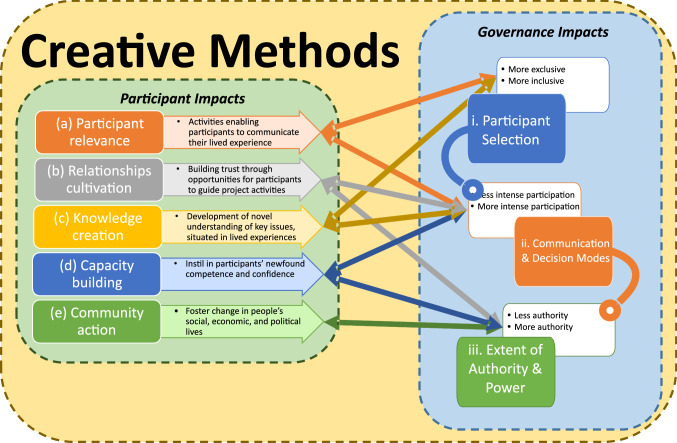
Theoretical frameworks for assessing Creative Methods (CMs) and their interconnections. On the left are the impacts for participants (after Hammond et al. 2018) versus the benefits for governance (after Fung 2006) on the right

Our findings explore these two dimensions of CMs’ transformative potential by evaluating (1) whether improvements to inclusion lead to a more equitable identification of problems and solutions, and (2) whether these novel solutions can be acted on by city decision makers, thus radically changing outcomes. To address these overarching questions, we have combined two complementary evaluation frameworks Hammond et al. (2018), and Fung (2006) (Fig. 1), to assess the potential of CMs for delivering urban transformations.

Hammond’s framework was specifically designed to assess the benefits of improved inclusion through arts-based engagement for indigenous communities (often categorised as vulnerable or excluded groups), and so is relevant for our analysis. It assesses inclusion across five themes: (a) engaging participants in relevant activities; (b) cultivating relationships of mutual trust, respect and power; (c) creating new (forms of) knowledge; (d) building individual or community capacities; and (e) initiating community action and change. To achieve transformation, improved inclusion requires a complementary governance system that is receptive and responsive. Fung’s framework assesses the links between participation and governance: who participates; how participants communicate to influence decisions; and how these discussions link with policy or public action. This is summarised as different ways of “*speaking, hearing, and exchanging information”.*

We have connected these two frameworks to assess the strengths and weaknesses of particular CMs in relation to their complementary transformational potential to overcome current practice and outcome shortfalls—namely, improved inclusion that shifts governance processes, leading to the identification of more equitable context-specific solutions implementable by policy and decision makers.

## Materials and methods

### Aims and context

Of all urban areas, cities and their decision-making processes have a great opportunity to re-direct urban design and investment into sustainable infrastructure that improves liveability for residents (United Nations Environment Programme 2011) whilst addressing poverty (International Council for Local Environmental Initiatives (ICLEI)—Local Governments for Sustainability 2018) and equity issues. This focus on developing sustainable pathways for cities is particularly pertinent to cross-cutting issues such as transport and mobility.

Mobility—being able to move—enables people to undertake their livelihoods, maintain social relations and be an active beneficiary of city living (Cuignet et al. 2020), thus avoiding disenfranchisement and exclusion (Lucas 2012). Infrastructures in the global south—particularly for mobility—are often broken, incomplete, badly regulated, underfunded and reliant on vernacular improvisations (Amin 2014) to make them function, let alone aspire to a level of liveability. Human vulnerability and resilience go hand in hand. Poor and vulnerable users have their mobility undermined and are forced to demonstrate resilience by using knowledge discovered through, often hazardous, lived experiences, and applying their imaginations to identify solutions to keep the city functional, if still risky and inequitable.

Road traffic crashes now cause up to 50 million injuries per annum and represent the eighth leading cause of death globally, claiming more than 1.35 million lives annually (World Health Organization 2018). For LMICs, this is a particularly pressing concern as road traffic fatalities are surpassing those due to HIV/AIDS, tuberculosis and diarrhoeal diseases. These impacts are particularly skewed towards the vulnerable: those who walk, cycle or rely on public transport, who make up most urban residents.

Nairobi, Kenya and Kampala, Uganda were the focus of our experiments due to their significant road traffic issues and poor infrastructure for non-motorised transport (NMT). The World Health Organisation (World Health Organization (WHO) 2019) estimates Kenyan road fatalities are 13 500 per annum. In Nairobi, the United Nations Environment Programme (UNEP) reports that pedestrians account for 65% of fatalities (Cummings and Obwocha 2018). In Uganda, there are 29 direct road traffic related deaths per 100 000 people, of which 39.5% are pedestrians and 5.8% cyclists (World Health Organization et al. 2018). During 2016, Kampala suffered 44% of all Ugandan crashes and 19% of all fatalities. Police attributed this high crash and death rate to reckless driving and congestion (United Nations Economic and Social for Africa 2018).

Delivering safe, sustainable and equitable mobility solutions for cities is therefore a key infrastructure challenge. Solutions that enable liveability and take into account the requirements of the poor, elderly, young and other vulnerable groups need to be a critical part of future city development (Rajé et al. 2018). Tackling transport and mobility will help deliver a range of SDG targets. These directly include SDG 3 on health (increased road safety), and SDG 11 on sustainable cities (access to transport and expanded public transport), alongside a still wider range of targets that can be indirectly linked (SDG 1 on poverty elimination, SDG 7 on energy, SDG 8 on decent work and economic growth, and SDG 9 on resilient infrastructure). LMIC cities have the transformative potential to leapfrog development pathways for infrastructure systems, bypassing previous mistakes embedded in older legacy urban environments.

### Research methods

#### Impact assessment framework

**Fig. 2:**
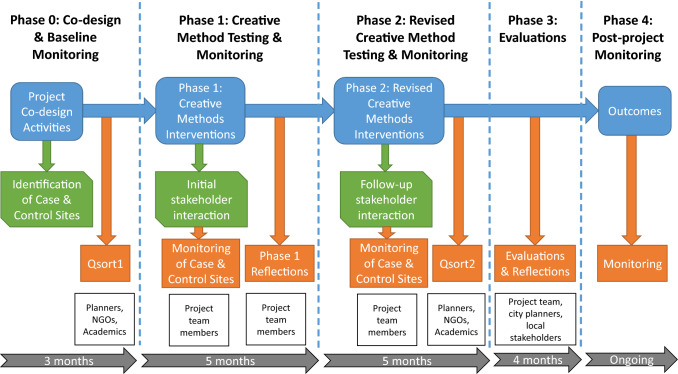
Overview of project phases (blue), activities (green) and outcomes (orange) undertaken by different participants (white)

Figure 2 illustrates the project phases and evaluation framework. In *Phase 0*, a real-world experiment to test a suite of CMs was co-designed with key individuals (planners, engineers, transport and stakeholder NGOs, business representatives), selected due to their official responsibility for urban transport or road safety, their responsibility for a specific case study site, or their representation of a particular constituency (vulnerable group, businesses or transport organisations). This group (hereafter referred to collectively as key experts) (see Supplementary Materials S1 for participants) worked alongside CM teams (comprising arts practitioners and academics) to identify suitable locations to structure the evaluation (see Table 1 (with S2 listing inclusion criteria)). To enable reflection on the additionality and unique contribution of CMs to outcomes, control sites were similarly identified with comparable mobility issues. This allowed comparison of CMs to ‘business-as-usual’ development processes more typically undertaken in each city.

**Table 1:** Intervention and control sites descriptions

Intervention (case) site	Justification	Control Site	Justification
I1—Luthuli Avenue, **Nairobi** (1°17′01.76″ S 36°49′40.70″ E)	**Context**: Complete corridor connecting town districts; Pedestrian walkway; Retail corridor; Business owners already receptive to change **Challenges**: Lack of provision for disabled users; Calling for intervention with high number of road users **Interventions**: Existing works on Accra road; Feeder road for Bus Rapid Transit (BRT) **Benefits:** High visibility; Might help urban regeneration of downtown Nairobi	Woodvale Grove, **Nairobi** (1°15′42.21″ S 36°48′12.38″ E)	**Context**: Part of a bigger connecting corridor to central business district **Challenges**: Traffic congestion **Interventions**: Existing interventions to pedestrianise planned; Interest from Nairobi County Council to inform design of BRT **Benefits**: High visibility scheme
I2—Killimani Ring Road (Ya Ya Junction), **Nairobi** (1°17′31.97″ S 36°47′14.04″ E)	**Context**: Diverse modal split and high number of users; Multiple public transport termini; Lack of provision for disabled users **Challenges**: Child unfriendly crossing; Accident hotspot **Interventions**: Re-designed road crossings with pedestrian reservation areas **Benefits**: High visibility; Interest from local community in improvements	Jogoo Road, **Nairobi** (1°17′28.02″ S 36°50′38.99″ E)	**Context**: Location used by multiple transport modes **Challenges**: Road safety issues; Congestion issues **Interventions**: Part of the BRT route; Existing planned interventions; Part of urban renewal programme **Benefits**: High visibility scheme; Enough space to accommodate multiple travel modes
I3—Upper Namirembe Road, **Kampala** (0°18′46.56″ N 32°34′44.01″ E)	**Context**: Public transport hub **Challenges**: Known engagement problem for local authority; Existing congestion; Road safety issues; Security improvement implications; Air and noise pollution improvements **Interventions**: Part of planned pedestrianisation scheme **Benefits**: High visibility of scheme; Outcome could be more efficient transport network	Lower Namirembe Road, **Kampala** (0°18′52.56″ N 32°34′21.23″ E)	**Context**: Public transport hub **Challenges**: None **Interventions**: Part of planned pedestrianisation scheme **Benefits**: Existing acceptance of planned scheme amongst stakeholders; Outcome could be more efficient transport network
I4—Bat Valley School Crossing, **Kampala** (0°19′21.03″ N 32°34′28.06″ E)	**Context**: Vulnerable road users at risk (school children) **Challenges**: Known road safety issue; Intractable problem **Interventions**: None planned **Benefits**: Approach could be replicable if successful	Comparator public school crossings, **Kampala** (0°19′10.44″ N 32°34′40.49″ E)	**Context**: Vulnerable road users at risk (school children) **Challenges**: Known road safety issue; Intractable problem **Interventions**: No interventions or improvements planned **Benefits**: Similar challenges to intervention site

For Phase 1, the key experts and CM teams identified specific suites of CMs to trial at each intervention site, described below in Table 2. CMs were selected for testing based on their perceived suitability for engaging targeted stakeholders (residents, school children, pedestrians, businesses, transport operators) alongside their ability to generate information relevant for the planning process. After five months of testing, the impacts of these CMs were evaluated using a mixture of collected monitoring and reflection data and were either expanded upon or replaced. The criteria for replacement were either that impacts from Phase 1 could be amplified through different methods; or that an alternative method would deliver an improved or complementary impact during *Phase 2* of testing. *Phase 3* focussed on co-creating final evaluations of the methods impacts with the key experts and CM teams engaged in Phases 0–2.

**Table 2:** Descriptions of tested CMs including benefits and challenges (as evaluated by our CM practitioners; project academics and key experts (academics, planners, NGOs, etc.)

**Creative method** *(D* = *Digital;* *P* = *Physical object/artefact/ event;* *M* = *Mixed D&P)*	**Description and testing locations** *I1—Luthuli Ave. Nairobi; I2—YaYa Junction Nairobi; I3—Namirembe Ave, Kampala; I4—Bat Valley School, Kampala. (Fully deployed method (F); Limited trialling of method (T))*	**Description of benefit and challenges (in italics)** ***Inclusion Benefits: Participants relevance (a); Relationship cultivation (b); Knowledge creation (c); Capacity building (d); Community action (e)*** ***Outcome Benefits:*** *Participant selection (level of Inclusion)—1i* = *State; 1ii* = *Professional and lay stakeholders; 1iii* = *Random selection to open self-selection; 1iv* = *Diffuse publics* *Communication and decision modes (intensity of participation)—2i* = *Listen as spectator; 2ii* = *Express or develop preferences; 2iii* = *Aggregate, bargain; 2iv* = *Deliberate and negotiate* *Extent of authority and power (level of authority)—3i* = *Personal benefit; 3ii* = *Communicative influence; Advise and consult; 3iii* = *Co-governance and direct authority*
Mine craft model (D)	Digital recreation of street allowing visualisation and participant modification of infrastructure (I1) (T)	Computer game-based visualisation targeted at engaging children (a,1ii). Enabled children to independently manipulate the virtual street, designing improvements on their own terms (c,2ii,3i). *Lack of IT resources restricted the methods full application beyond a limited trial*
On-street photographs (M)	Documentary photography of conditions at sites for different users (I1,3) (F)	Photographs captured lived experiences of street users (a,1ii). This increased shared understanding between photographers (in I1 mainly University students new to the sites) and street users through in situ conversation (b,2i)). Images used at feedback events (2i) stimulated conversation further widening learning (b, e, 3i). *Limited number of active participants*
Drone imagery (D)	Highlighting unsafe interactions of road users at different times of day to aid understanding (I2) (T)	Captured road safety issues (c,3ii) and showed potential to be further assessed using automatic image analysis to quantify risks or artistic interpretations (sonification, etc.). *Significant* a*dditional time and resource would be required to fully realise these supplementary benefits*
Virtual reality (VR) streetscapes (D)	Enabling virtual experience of road safety hazards to aid understanding (I2) (T)	Increased shared understanding (2i) of pedestrian experience (c, 1ii) including for key governmental decision makers (3ii). Novel for stakeholders so stimulated new interactions but *whether learning is improved over wearable cameras would require further analysis*
Social media content (D)	Discussions on road safety and mobility issues (I1,2,3) (F)	Twitter and WhatsApp enabled users to share their project experiences (a,1iv,2i) stimulating online dialogues (3ii). *Impacts from these interactions requires further assessment to effectively ascertain transformative benefits*
Wearable cameras (D)	Capturing the lived experience of street users including road safety risks (I1,3) (F)	Recording first person experience (a, b) allowed street users (1iii) to capture and communicate road space hazards (close passes with vehicles; poor infrastructure) (c,2i,3ii). *Issues of personal safety and anonymity bring some methodological challenges*
Participatory GIS maps (M)	On-street in situ physical mapping of mobility issues digitised and available online (I2, 3) (F)	Passers-by (1iii) identified locations and perceptions of road hazards and safe spaces ((a, b, c); these data informed the development of infographics and pop-ups (2ii) to incorporate community knowledge (3i). *Challenge was achieving representative sample of users*
Infographics (D)	Detailing the statistics on road safety and the revealed preferences for improvement from other engagement events (I2) (F)	Used at multiple feedback events to stimulate discussion (c,1iv). Improved knowledge exchange and shared understanding (2ii) boosted capacity for community led change (d,e,3i). *Challenge was selecting which data to represent without undue bias*
Urban dialogue discussion workshops and online forums (M)	Series of interlinked public discussion meetings held in city centre venues (I1, 2) (F)	Open invite public discussion meetings (hosted in person (1i) and online (1ii, a)) using CM outputs from on-street photographs, participatory-mapping, etc. to present findings and gain feedback (b,c,2ii,2iii). Built shared understanding and discussion built community confidence to ask for change (d,e,3ii). *Challenge was participants tended to be the interested and educated; not vulnerable or street users*
Digital storytelling (D)	Curation and online dissemination of user stories to illustrate road space issues including footage from wearable cameras and photographs (I1,2,3) (F)	Utilised outputs from other CMs to represent participants' stories (a), to more effectively communicate (b,3i) their lived experience and generate further reflections (2ii), feedback from viewers (c,2ii), and stimulate action (e). *Outputs require promotion to key decision makers to achieve transformational impacts*
Time-lapse videos (D)	Time series videos documenting the changing use of street space (daily) and pre- and post-on-street interventions or improvements (I1) (F)	Effectively engaged planners (1ii) and stimulated debate at feedback events (3ii). Imagery could be further analysed to assess vehicle, pedestrian activity pre- and post-providing quantitative evidence (2i). *Significant* a*dditional resource would be required to fully realise these supplementary benefits*
Urban guerrilla signage (P)	Doctored creative road signs highlighting safety issues to street users (I2) (F)	Developed from PGIS data and community feedback. Designed to disrupt road users’ actions (1iv,2i) making them consider road space and safety in a different way (d,e,3i). *Challenges are obtaining permissions and monitoring impacts, especially from motorised transport users*
Theatre and performance (P)	Interactive theatre performances of road safety issues with schoolchildren (I4) (F)	Interactive performance involving children (a,3i) role-playing road safety actions and learning (b,c,d,e,2i,3i). Assessed using follow-up visits after activity. *Challenge is bringing to scale to achieve city-wide impacts*
Creative play (P)	Using play including songs to simulate safe and unsafe road crossing for children (I4) (F)	Interactive performance involving children (a,3i) role-playing road safety actions and learning (b,c,d,e.2i,3i). *Challenge is bringing to scale to achieve city-wide impacts*
Comics and cartoons (P)	Co-designed comics detailing road safety issues and messages for school children (I4) (F)	Blank comics designed to be coloured in by children (a,1iv) detailing road safety messaging stories (d,e,2i,3i). Effective, cheap and easy to deploy. *No significant drawbacks identified but may only suitable for younger children*
Street art canvases (P)	On-street engagements using painting to capture road safety experiences and visualise proposed improvements (I3) (F)	Highly interactive (a) with CM practitioners interacting with participants (1iii) to visualise their experiences and identify solutions (c,d,e,2ii). Effective at reaching non-literate and street users. Easy to disseminate outputs widely online (d,e,3i). *Time-consuming to enable different views to emerge*
Design competition (D)	Engagement of University students to develop plan for road improvements responding to the co-created design brief (I1) (F) (see below)	Using preferences distilled from multiple CM outputs a design brief was generated (b). This was utilised in a design competition for University student teams (1ii) to develop infrastructure and road layout solutions targeting safety and sustainability (d,e,2iii,3ii)
Pop up feedback displays (P)	Digital and on-street/in situ visualisations of project outputs for dissemination back to participants (I1,2,3) (F)	Visual nature of project outputs engaging for participants (a,b,1iv). Improving their understanding of different perspectives (c,2i), stimulating dialogue that increased awareness of issues and solutions (d,3i) promoting action (e). *Impact limited by location and participant availability*
Placemaking on-street events (P)	On-street events including temporary partial closing and re-imagining of street space (I1,3) (F)	Allowing opportunities (a) for all street users (b,1iv) to explore how an improved road might function in reality (c,2ii) and feedback on project CM outputs (d,e,3ii). *Disruptive and requires official permissions so can be expensive*
On-street architectural models (P)	3D models deployed in situ on-street enabling interactive planning with street users to co-create a design brief for a safer streetscape (I1) (F)	Engaging for participants (as evidenced by high footfall, 800+) (a,1i). Visual, interactive nature allows non-literate to comment (b,c,2ii). Provided useful information to input into other CMs (3ii)
3D zebra crossing (P)	Mobile temporary intervention to highlight road crossing safety issues to drivers and pedestrians (I3) (F)	Intended to be disruptive for road users (d,1iv) encouraging them to rethink safety issues (e,3ii). *Generated considerable interest (media, politicians) but would need to be part of wider mixed-methods campaign to maximise impacts or be deployed strategically to avoid fatigue*

**Table 3:** Transitions in the planner’s perspectives on creative methods and inclusive planning (Q-statement numbers [S#])

Factor 1	Factor 2	Factor 3
**Q-Sort1—Pre-project**		
**Mass transit futures** **Overall summary** Sees shortfalls in current engagement around planning and believes NMT should receive greater emphasis in city road space plans—rather than cars *Planning Perspective* Believes in open inclusive engagement [S10] but thinks existing approaches currently don’t allow for this [S13]	**Congestion smasher** **Overall summary** Wants to widen planning engagement to identify congestion reducing solutions for a mixed mobility future *Planning Perspective* Strongly believes in the need to widen engagement opportunities [S5] with the aim of identifying solutions to congestion [S1]	**Inclusive planning is the answer** **Overall summary** Wants planning reforms to improve engagement, using mixtures of approaches to get greater inclusion *Planning Perspective* Believes that current planning is not effective at representing all users [S18, 19,23] instead focussing upon engineers’ solutions and car drivers [S9,31,32] and should become less formal [S28]
**Q-Sort2—Post-project**		
**More inclusive planning for a car-free future** **Overall summary** Pro-NMT and public transport, and anti-car with a belief that current planning approaches are ineffective *Planning Perspective* Existing approaches are ineffective for engagement [S10,13] and more creative approaches are needed [S8]	**Creative congestion smashing** **Overall summary** Planning is critical and should be improved with creative engagement. Informal transport should be restricted to promote walking and reduce congestion *Planning Perspective* Planning is critical for city development [S33]. Current non-inclusive [S31,32] engagement is ineffective and could be improved with creativity [8] but full representation is not essential [S23]	**Inclusive creative planning is the answer** **Overall summary** Better community engagement would improve planning and creative approaches could achieve this to improve walking options *Planning Perspective* Local community engagement is key [S12] with informal events being effective [S28]. Creative methods would widen engagement [S8, S20] making them more representative [S23]

#### Monitoring and evaluation of change data collection and analysis

As the intervention period for Nairobi and Kampala was relatively short (approximately 12 months), a mixed-method evaluation was used (quantitative data on inclusion and qualitative data on outcomes—detailed below), as it focusses on immediate changes for particular stakeholder groups (Rockwell and Bennett 2004). Individual CM activities were monitored continuously during Phases 1 and 2. Outcomes were assessed internally by the CM team (academics and CM practitioners) during Phases 1 and 3. A wider evaluation was undertaken at Phase 4 with key experts from Phase 0 and CM activity participants (e.g. Bat Valley teachers) during two evaluation workshops (one face-to-face in Kampala; and the other virtual).

#### Inclusion evaluation

Evaluation reflections on inclusion (supported by feedback recorded in impact stories, videos, social and print media from events or content received from stakeholders, including local users of the case study sites) have been scored by the academic project team to assess impacts on widening inclusion. They were scored using the metrics connected to Hammond’s framework, specifically, levels of participation (number of participants; or numbers of social media messages) (linked to (a) and (d) of the framework); engagement outcomes (linked to (b), (d) and (e)); and the types and number of outputs generated (e.g. number of maps, variety of participants) (linked to (c)).

#### Outcome evaluation

For CM approaches to be transformative in terms of outcomes and mainstreamed within infrastructure development, they need to be made salient to key decision makers (Dolan et al. 2012; Abson et al. 2017). Salience is influenced by making something tangible and personal, and is reinforced through social interactions with others who then support emerging belief norms (Pelling et al. 2015). Salience was assessed using a combination of Q-sort (Alderson et al. 2018) supplemented by Most Significant Change (MSC) stories (Davies and Dart 2005; Wilder and Walpole 2008). Additional outcome evidence came post-interventions (Phase 4), when real changes on the ground had been monitored (relating to (e) in the theoretical framework).

The concourse of Q-sort statements was developed from various sources (see Supplementary Material S3) with thirteen statements representing beliefs on engagement in planning. Sort exercises pre- and post-interventions were undertaken with a subset of Phase 0 key experts to reveal their underlying subjective beliefs (Cuppen et al. 2010). Factor analysis identified belief groupings and changes in statement weightings, revealing shifting beliefs after interaction with CM outputs amongst the individuals responsible for planning and infrastructure.

MSC stories from key experts and activity participants described the influence of their encounter(s) with CMs, allowing them to make sense of these experiences in their own terms and language. The MSC narratives focussed on our particular interventions and CMs, but put them into the context of other factors beyond our control influencing the outcomes (McClintock 2004). Narratives collected from control sites provided contrast, revealing the inclusion and governance benefits of utilising CMs. MSC stories were coded using qualitative research software (NVivo) to identify additional governance benefits or shortfalls that CMs brought, which would not have occurred through conventional engagement (Baú 2019).

## Results

### Methods categorisation

**Fig. 3:**
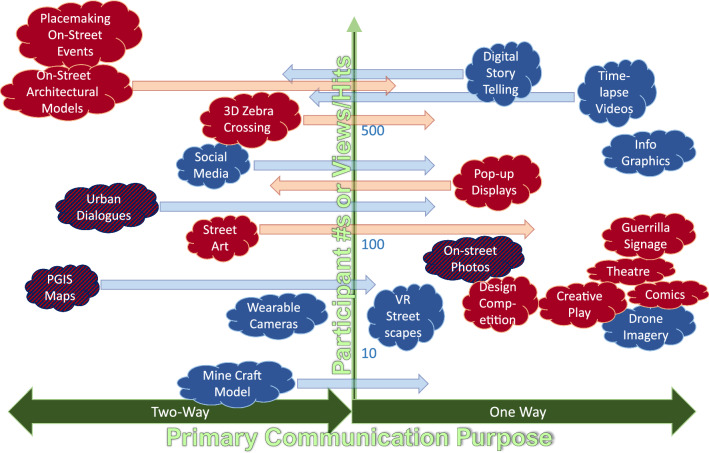
Overview of tested CMs, participation rates and communication purposes. Arrows indicate mixed communication purposes, potential and scale. (Blue—Digital; Red—Physical; Hatched—Mixed delivery (digital and physical)). Numbers of people engaged are participants or viewers accessing digital content

For comparison, the trialled methods have been categorised into their *delivery* mode (either digital or analogue, i.e. physical events or objects) and *purpose* (either one-way outreach and feedback or two-way knowledge exchange and dialogue) (Fig. 3, Table 2).

### Inclusion impacts

Qualitative analysis of the key expert and CM practitioners’ reflections revealed interrelated emergent themes around inclusion*.* The longitudinal impacts on decision-maker beliefs of inclusion changes were assessed through the Q-sort findings.

### Generating new knowledge and engaging marginalised voices

CMs were described as useful in generating new information and engaging typically excluded or hard-to-reach groups which key experts (particularly engineers) found useful for rebalancing spatial justice (Soja 2010) debates on road space allocation. There are… *“two sides to transport—the influential—the car users, businesses and matatu* [mini-bus taxi] *owners. On the other side the silent majority—the walkers and commuters who use public transport. We need to shift the focus to the silent majority” (Quote from Urban Planner, [UP]).* We… “*need to design for what people want—rather than thinking we already know what they want” (Quote from Engineer [E]).* Greater inclusion facilitated by CMs was viewed as critical for urban development by ensuring that transport was made *“efficient—so it can also tackle big challenges such as climate change” [E].*

CM practitioner reflections identified ethical and context concerns, recognising the need for care in methods selection. For example, on-street approaches did not reach passing drivers, leading to exclusion (however, digital methods might). Digital storytelling required modifications to ensure the anonymity of vulnerable participants, and on-street photography needed permits to overcome security concerns.

Key experts’ feedback in Phase 4 revealed they believed CMs had led to improved infrastructure planning and road safety awareness. Bat Valley School Teachers commented that *“when you just sit and talk to someone they can easily forget, even as adults. But the methods used of drawing and painting, it aids their memory, so children will remember those messages to keep themselves safe”.*

The need for improved planning engagement was highlighted by the Nairobi CBD comparator site findings. Here a road widening scheme had displaced informal traders. Only a subset of traders had been consulted prior to demolitions. Impacts on traders were mixed, with the majority displaced with unknown livelihoods outcomes, whilst a minority had relocated to a private market with greater rent overheads. Meanwhile, the mobility, safety and sustainability of the road improvements had been undermined by unconsidered (and unconsulted) driver behaviours who reoccupied and re-used the widened road margins as a vehicle park. A UP commented *“is the solution we designed what the users want?”*.

### Improving group interactions

CMs’ immediacy and visuality were particularly beneficial in improving shared understanding. Visual and tactile methods enabled greater engagement equity, particularly from non-literate groups, by enabling them to reveal their own reality via stories or images of lived experiences. For example, they *“enabled matatu drivers to communicate using their language. We need to learn and engage them on that level so that we can include them in the changes in the city [E]”.*

The versatility of the approaches was praised; *“what really stood out for me were the range of creative methods that were available to consult with local people… options from digital storytelling to drones… You can pick the right methods for the audience that you want to engage” [Quote from International Agency Spokesperson].* This variety could help overcome problems of participation fatigue with conventional engagement.

### Impacts on decision makers

Reviewing the Q-sort factor analysis revealed three distinct viewpoint groupings (Table 3) both pre- and post-project. Focussing only on the differences in statements related to planning, the variation in views across time periods between these groups is subtle. However, an emerging trend was that after exposure to CMs, participants agreed more strongly that these new methods could be effective in widening inclusion, thereby benefiting planning processes. This universal acceptance was irrespective of their ultimate city development beliefs (see Supplementary Material S3 for Q-sort scores).

Comparing changes in the planning-related statement weights between cities indicates problems with conventional planning were more pressing in Uganda. Opinion shifts in Kampala were significantly stronger than those in Nairobi (*t*(28) = 2.93, *p* = 0.003). This may be indicative of different policy goals or underlying engagement priorities between planners in the two capitals.

**Fig. 4:**
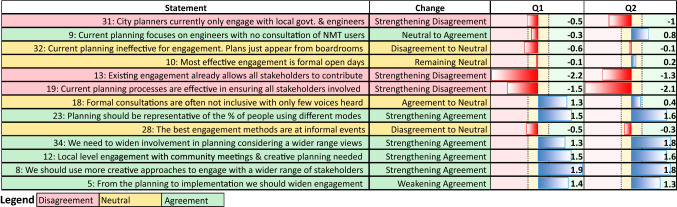
Mean Q-sort statement scoring at timesteps Q1 and Q2. (Red bars indicate negatively scored statements; blue bars positively scored statements, with zones indicating the level of agreement, disagreement or neutrality of views)

Comparing sorts longitudinally (Fig. 4), there is a realisation that current engagement practices are not representative or effective [Statements 31, 9, 32, 13, 19, 18]. This evidence of an emerging shift in views is reinforced by increasing agreement of the need to widen engagement and inclusion [S23, 34, 8, 5]. Critically, the results indicate increasing agreement amongst key experts that CMs have a useful role to play in achieving wider inclusion in urban planning issues [S12, 8].

**Fig. 5:**
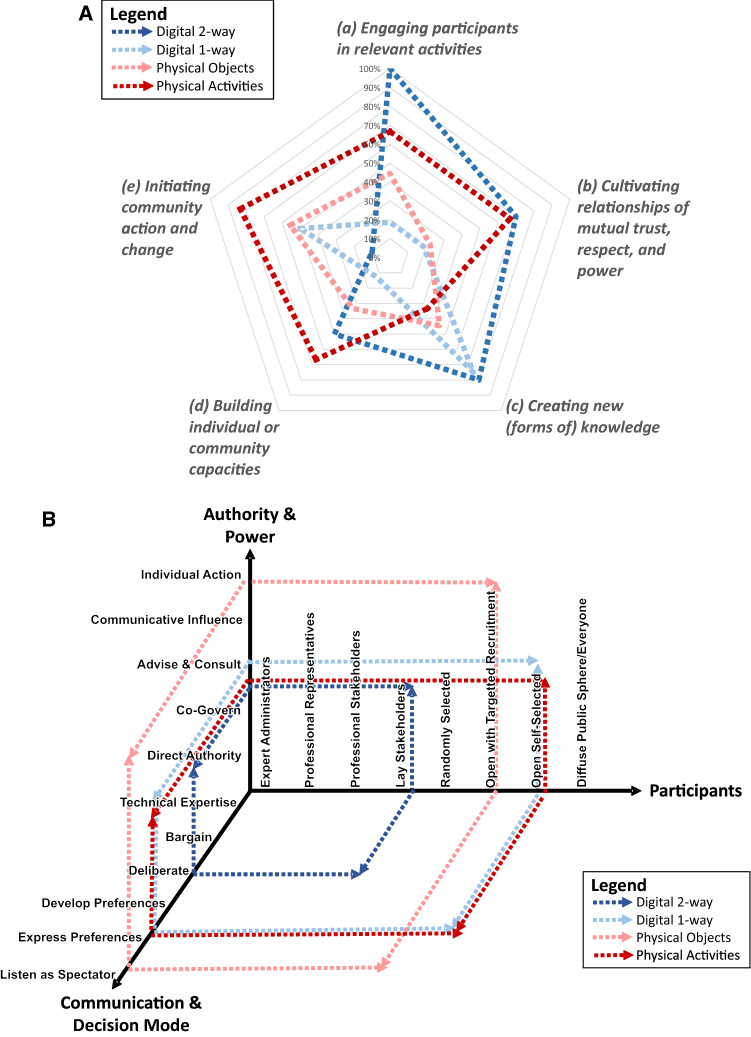
Assessment of the participation benefits (**a**) (after Hammond et al. 2018) and governance impacts (**b**) (democracy cube after Fung 2006) for digital vs. physical CM

Our inclusion evaluation of CMs (levels of participation; engagement outcomes; the types and number of outputs generated) revealed differences in participation quality (see S4 for individual methods scoring). Digital approaches are highly relevant when enabling the creation of new knowledge from a wide cross section of citizens (crowdsourcing), with a caveat on equity issues (Tanui 2018). Analogue on-street activities are most useful for building community capacity and stimulating bottom-up actions. Our key finding is that due to these differing strengths and weaknesses of individual approaches, only deploying a mixture of complementary CMs can deliver significant improvements for inclusion (Fig. 5a).

### Improved outcomes

#### Governance impacts

Planning engineers described how CMs could ensure that people (‘the software’), who use city infrastructure (‘the hardware’), understood the purpose of road safety improvements. CMs *“humanise the narrative” [E]* changing *“the way we interact with communities. We are now using more creative methods to communicate, including videos and graphics” (Policy Maker [PM])*, highlighting enthusiasm for one-way outreach. For improved two-way communication and learning outcomes *“awareness needs to flow both ways between engineers and public [E]”* and “*planners need to include creativity in their thinking—so that they plan for the context of the real city, not an abstract place” [UP].* The implications of differing enthusiasm for communication versus dialogue form part of our discussion.

#### Building trust and relationships

CMs better sensitised users to the purpose and benefits of planned solutions, helping to dispel local opposition. In Kampala, the CBD site (I3) had resistance from local businesses and street users, who opposed a proposed traffic calming scheme. By utilising CMs, the potential benefits (road safety, business revenue and environmental quality) were communicated more equitably and effectively. This improvement in shared understanding built a more trusting relationship between the stakeholders (mediated by the project team). By the end of the project, the road improvement construction was underway without significant protest, somewhat to the surprise of the city planners. In Nairobi, the Matatu owners and drivers were successfully engaged in co-designing and agreeing improvements to Luthuli avenue (I1), enabling the implementation of improvements.

Scoring governance impacts reveals more subtle variation (Fig. 5b) compared to that for inclusion. Digital one-way outreach can inform a wide range of citizens on plans and decision processes, whilst analogue approaches enable focussed inclusion. However, this is typically in a specific site, as the audience has to be able to physically witness the activity or artefact to benefit. In relation to the intensity of participation in decision making, CMs at best enabled the development of preferences, or allowed citizens to view processes as spectators. In this regard, digital approaches facilitated a marginally greater degree of interaction than on-street engagements. Finally, findings in improvements in power relationships indicate that, for infrastructure development, CM outputs communicate the lived experiences of users, thereby influencing the planning outcome tangentially through an improved understanding of official agencies. However, our tested CMs suite did not enable direct influence on outcomes, as may be the case for other approaches aimed specifically at rebalancing power, like a citizen jury, for example (Fung 2006). Critical differences between digital and physical methods were in the specificity of outputs, with digital generating wider inclusion but generic ideas, whilst physical revealed place-specific detailed solutions for further deliberation.

#### Post-project outcomes

**Fig. 6:**
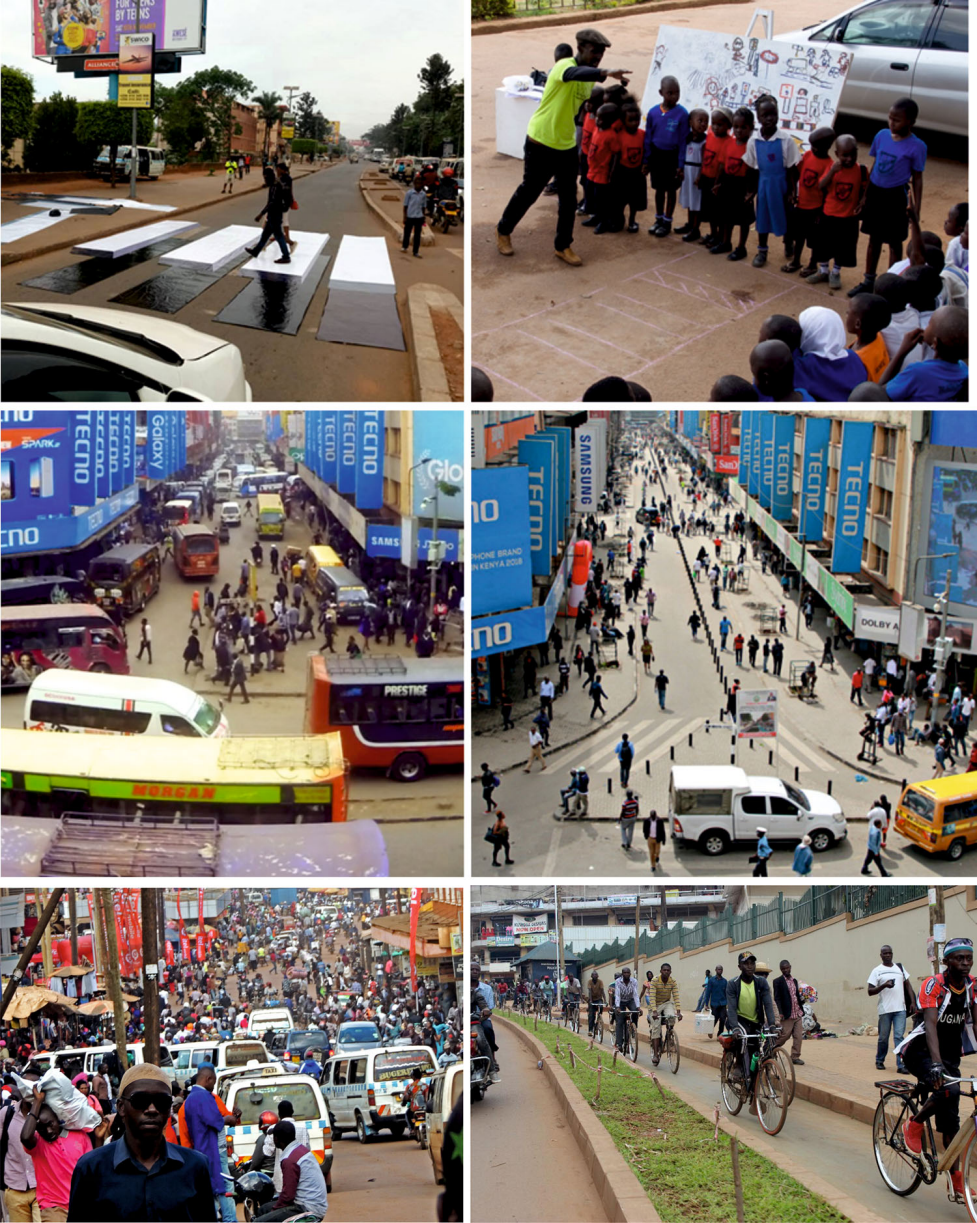
Examples from our interventions. Top Left—3D zebra crossing in Kampala; Top Right—creative play teaching road safety in Kampala; Middle Left—Luthuli Avenue pre-traffic calming; Middle Right—after scheme implementation; Bottom Left—Namirembe Road pre-improvements; Bottom Right—after improvements with segregated walking and cycle lane

From a behaviour change perspective (Dolan et al. 2012), our results indicate our co-design, CM outputs, and ultimately outcomes were salient and useful for transport professionals. This new awareness led to requests to embed the approaches within government guidance. *“We need to institutionalise* […CMs…]. *Have a package for government authorities so that they can modify the structure of their work” [UP]. “As we develop our national policies, we can bring in these creative approaches alongside more traditional methods” [PM].* There was enthusiasm for future use of the trialled methods (e.g. 3D zebra crossing (Fig. 6)) by Kampala Capital City Authority (KCCA) officials. A six months follow-up investigation into children’s road safety knowledge (Mwesigwa 2019) supported a positive assessment of learning outcomes. Education heads from neighbouring schools responded on social media to request access to the road safety training, indicating how digital approaches can widen engagement and increase demand for CMs. KCCA has indicated that the road safety activities would ideally be rolled out to all schools (budget allowing) (Mwesigwa 2019). In terms of impacts for vulnerable users, in both cities’ traffic calming schemes, measures co-designed with CMs have been successfully implemented, supporting non-motorised mobility options (as evidenced by high usage of cycle lanes under COVID-19 in Kampala) and reducing air pollution [with UN Environment weekday measurements on Luthuli Avenue showing an average reduction in PM_10_ of 52% from 87.8 to 41.9 µgm^3^ (Pers. Comm.)].

## Discussion

Novel creative approaches could make contributions to solving a range of wicked urban problems around equitable infrastructure planning and behaviour change. CMs can actively harness innovation, contributing to resilience building (Ernstson et al. 2010) that stems from diversity and redundancy in systems (Ernstson and Barthel 2010; Elmqvist et al. 2019), thereby aiding transformative processes. CMs can reveal functional vernacular improvisations explicitly, highlighting how different stakeholders’ behaviour will interact with changed environments. CMs could also cooperatively identify governance rules for the use of spaces, when understanding these behavioural dimensions are as critical to success as the quality of the built infrastructure. Currently, a lack of clarity on behaviours often results in ‘solutions’ being undermined, because they do not service the excluded majority. Incorporating a consideration of probable behaviours into infrastructure plans could result in improvements in practice more closely operating in line with planners' intentions (Nikulina et al. 2018).

### Transformative potential and SDG delivery

Three key governance challenges identified for the SDG delivery include (i) cultivating creative action by creating inclusive decision-making spaces; (ii) making trade-offs to achieve equity; and (iii) accountability of decision makers in relation to outcomes (Bowen et al. 2017). Our evidence indicates that greater adoption of CMs could make a worthwhile contribution to addressing issues of inclusion (i) and equity (ii), helping ensure the effective localisation of the SDGs to specific places. By enhancing the inclusion of vulnerable communities, CMs could assist in delivering robust, equitable development plans, meeting an element of the ambitions for transformative change embedded in the SDG agenda. Our evidence indicated that city planners began to appreciate that greater inclusion could deliver solutions that met both a wider cross section of residents' needs and, critically, were also practical to implement. This implies they valued CMs for their pragmatic utility in helping derive solutions over conventional engagement. However, there were also indications that some valued CMs purely for outreach benefits: to inform, co-opt and placate communities in relation to change, running counter to a transformative agenda. This particular risk is apparent as the methods adopted are primarily for 1-way communication rather than 2-way co-design or co-creation. Our Kampala intervention (I3) highlighted this dimension as we evaluated the benefits of using CMs to inform stakeholders on a pre-existing scheme, rather than generating bottom-up alternatives. Ultimately, the wider acceptance of the proposed scheme was greatly appreciated by the planners and communications team in KCCA but highlights the risks of CMs placating stakeholders, rather than enabling planners to address genuine concerns.

### Limitations

Significant pre-existing conventional engagement, sensitising stakeholders to the issues, had been undertaken where we observed notable successes. The project team also operated independently of official planning departments, helping generate a trusting environment. Whilst our case studies included elements of conflict, we did not explicitly test the methods efficacies for overcoming disagreements, although CMs have been successfully utilised in this role (Premaratna and Bleiker 2016; Zournazi 2018) in other contexts.

Further CMs’ experimentation is required to evaluate impacts when used by formal agencies, where no prior groundwork has been undertaken, and around explicit conflict resolution issues. This testing could include exploring ethical dimensions, such as the issue of CMs revealing illicit behaviours. Whilst anonymity can sometimes be maintained, CMs’ strength of increasing shared knowledge may also be problematic for certain settings or when used by official agencies. Further research is required to explore the possibility of planners valuing these approaches only as a way of co-opting publics to coerce widened acceptance of top-down plans. Finally, transformations are known to be long processes, so to fully assess the impacts that CMs could generate within urban systems (including further catalysing change in institutions, investments and governance), longer timespans or a greater intensity of activities would be required.

To achieve urban transformations, a significant shift in the operational norms, goals and resource flows of institutions towards more sustainable pathways is required. This represents a greater challenge than inclusion and co-design, and is one which CMs may only superficially address. For example, whilst we improved children’s road safety skills to reduce risk, we did not address the cause of the hazards: the prevalence of motorised vehicles supported by infrastructures and driver behaviour. We also did not instigate changes in the structures of city councils planning bodies. If CMs could induce a widening of inclusion and outcomes, such wider transformations might emerge over time, due to demands from groups who are currently excluded from official decision-making structures. Exposure to CM processes amongst key stakeholders may act as a catalyst for this fundamental shift. Without such structural changes, CM-facilitated improvements in inclusion and outcomes alone will simply enrich planning processes, falling short of transforming cities and missing the opportunities embodied in the SDGs.

## Conclusion

Our key findings reveal that using a complementary mixture of CMs can enable typically excluded users to contribute effectively to planning processes. CMs can improve group interactions, leading to a greater commonality of shared understanding between stakeholders. Practical exposure to these methods begins to change planners’ understanding of the role and benefits of engagement. Compared to the efficacy of public meetings or focus groups (Fung 2006), our evidence indicates that CMs bring significant improvements in terms of the diversity of participants; ways of exchanging information; and different levels of empowerment, contributing towards overcoming planning (Nordström and Wales 2019) deficits. Risks include CMs being deployed purely for outward communication to co-opt communities into official schemes and the ethical challenges of revealing illicit behaviours of stakeholder groups to officialdom. However, if used for inclusive dialogue, increasing CM use could contribute to improving direct citizen participation in policy-making, and aligning outcomes with those of the wider public, to enhance legitimacy and offset governance failures (Fung 2015). This connects CM use to the normative, substantive and instrumental dimensions of justifications for participation (Blackstock et al. 2007), which underlie many of the SDGs. Citizens who are affected by urban challenges are well placed to provide information relevant to devising novel solutions or identifying the unconsidered challenges of proposed infrastructure. CMs enable a greater cross section of people to provide this information, highlighting explicitly the complexity of mobility challenges more transparently and in a form that enables and empowers dialogues, thus helping to build resilience (Adger et al. 2020). CMs empower citizens, helping them to generate their own bottom-up solutions to problems, and enable equitable co-production, leading to transformative change.

## Supplementary information

Below is the link to the electronic supplementary material.

(PDF 1259 kb)
