# Supplementary material for: Transforming urban planning processes and outcomes through creative methods
Source: Ambio. 2021 Feb 14;50(5):1018–34. doi: 10.1007/s13280-020-01436-3 (PMC7882470; doi:10.1007/s13280-020-01436-3)
Supplement: Supplementary file 1 — (PDF 1259 kb) [file 13280_2020_1436_MOESM1_ESM.pdf]

Ambio
Electronic Supplementary Material
This supplementary material has not been peer reviewed
Title: **Transforming urban planning processes and outcomes through creative methods**
Steve Cinderby, Annemarieke de Bruin, Howard Cambridge, Cassilde Muhoza, Amanda Ngabirano

Supplementary Materials S1

Project Participant Descriptions

Location(s)	Participant Type	Count
Kenya	Academic	6
Kenya	Creative Practitioner	8
Kenya	Governmental	7
Kenya	NGO	9
Uganda	Academic	5
Uganda	Creative Practitioner	9
Uganda	Governmental	7
Uganda	NGO	4
Uganda & Kenya	Academic	4
Uganda & Kenya	Creative Practitioner	4
Uganda & Kenya	Governmental	2

Project Participant Descriptions

Key to case study locations: I1 - Luthuli Avenue, Nairobi; I2 - Killimani Ring Road (Ya Ya Junction), Nairobi; I3 - Upper Namirembe Road, Kampala; I4 - Bat Valley School Crossing, Kampala

Key to Participant Type: A = Academic; C = Creative Practitioner; G = Governmental; N = Non-Governmental NGO)

Loca-tion(s)	Case Study	Organisation	Role in Project	Type
Kenya	I1 & I2	University of Nairobi	Urban Planning & Transport Expert	A
Kenya	I1 & I2	University of Nairobi-Centre for Urban Research and Innovation	Urban Planning Expert	A
Kenya	I1	Technical University of Kenya		A
Kenya	I1	Architectural Association of Kenya	Urban Design Expert	A
Kenya	I1	Freelance	Street photographer	C
Kenya	I1	Freelance	Urban Design	C
Kenya	I1	Freelance	Urban Design	C
Kenya	I1	Freelance	Urban Design	C
Kenya	I1	Freelance	Landscape Architect	C
Kenya	I1	Pamoja Road Safety Initiative,	Educational illustrator	C
Kenya	I1 & I2	Freelance	Creative Methods Expert	C
Kenya	I1	Placemakers	Urban planner - Leading Case Study I1	C
Kenya	I1 & I2	Metropolitan Area Transport Authority (NAMATA)	Transport Engineer	G
Kenya	I1 & I2	Metropolitan Area Transport Authority (NAMATA)	Transport Engineer	G
Kenya	I1 & I2	Nairobi County Government- Roads, Public Works and Transport Dpt	Transport Engineer	G
Kenya	I1 & I2	Nairobi County Government- Urban Planning Dpt	Planner	G
Kenya & Uganda	I1, I2, I3, I4	UN-Habitat, Urban Mobility	Transport Expert	G

Kenya	I1, I2, I3, I4	UN Habitat	Urban Policy Expert	G
Kenya	I1 & I2	Kenya Urban Roads Authority (KURA)/NAMATA	Transport Engineer	G
Kenya	I1 & I2	Matatu Owners Association	Business Representative	N
Kenya	I1 & I2	Institute for Transportation and Development Policy (ITDP) Nairobi	Transport Specialist	N
Kenya	I2	Nairobi / Naipolitans	Urban planner/Blogger	N
Kenya	I1	Hope Raisers Initiative	Performing Artist	N
Kenya	I1	Nairobi City County Safer Nairobi Initiative and UN Safer Cities Project	Community Engagement Expert	N
Kenya	I1 & I2	Environmental Compliance Institute	Environmental Impacts Expert	N
Kenya	I1 & I2	Pamoja Road Safety Initiative	Director	N
Kenya	I1	Hope Raisers		N
Kenya	I1 & I2	Institute for Transportation and Development Policy (ITDP) Nairobi	Transport Specialist	N
Kenya	I1, I2, I3, I4	SEI	Urban Planning Expert	A
Kenya	I1, I2, I3, I4	SEI	Social Scientist	A
Uganda	I3 & I4	Makerere University	Transport Planner	A
Uganda	I3 & I4	Makerere University	Urban planning academic	A
Uganda	I3 & I4	Makerere University	Traffic and transport infrastructure academic	A
Uganda	I3 & I4	Makerere University	Urban planning academic	A
Uganda	I3 & I4	Victoria University	Urban governance and planning academic.	A
Uganda	I3 & I4	Kamapala (The Observer/The Guardian)	Journalist	C
Uganda	I3 & I4	Freelance	Designer	C
Uganda	I3 & I4	Freelance	Artist	C
Uganda	I3 & I4	Crossroads Digital Multimedia	Animator	C
Uganda	I3 & I4	Freelance	Visual Artist	C
Uganda	I3 & I4	Freelance	Community Engagement	C
Uganda	I3 & I4	Freelance	Artist	C
Uganda	I3 & I4	Freelance	Film Maker	C
Uganda	I3 & I4	Freelance	Artist	C
Uganda	I3 & I4	Ministry for Transport - Kampala Govt of Uganda	Commissioner planning	G
Uganda	I3 & I4	Kampala Capatial City Authority	Head Public and Corporate Affairs	G
Uganda	I3 & I4	Uganda Road Fund (URF)	Chartered Civil Engineer/Transport Planner	G
Uganda	I3 & I4	Kampala Capatial City Authority	Planning directorate director	G
Uganda	I3 & I4	Ministry of works and transport Uganda	NMT coordinator.	G
Uganda	I3 & I4	Kampala Capital City Authority	Planner	G
Uganda	I3 & I4	Kampala Capital City Authority	Traffic engineer	G
Uganda	I3 & I4	Hope of Victims	Road safety team leader of Traffic Accidents (Hovita).	N
Uganda	I3 & I4	Uganda Sustainable Transport Network	Project manager Teens	N
Uganda	I4	Buganda Road Primary School	Teacher (40 pupils engaged)	N
Uganda	I4	Bat Valley Primary School	Teacher (100 pupils engaged)	N

Uganda & Kenya	I1, I2, I3, I4	ESP, UK	Transport Consultant	A
Uganda & Kenya	I1, I2, I3, I4	SEI	Social Scientist	A
Uganda & Kenya	I1, I2, I3, I4	SEI	Communications Expert	A
Uganda & Kenya	I1, I2, I3, I4	SEI	Monitoring & Evaluation Expert	A
Uganda & Kenya	I1, I2, I3, I4	Loughborough University, UK	Academic	C
Uganda & Kenya	I1, I2, I3, I4	Loughborough University, UK	Academic	C
Uganda & Kenya	I1, I2, I3, I4	Consultant	Visual Storyteller	C
Uganda & Kenya	I1, I2, I3, I4	Invisible Flock, UK	Visual Artist	C
Uganda & Kenya	I1, I2, I3, I4	UNEP	Transport Specialist	G
Uganda & Kenya	I1, I2, I3, I4	UNEP	Road Safety & Mobility Planning Policy Expert	G

Supplementary Materials S2

Figure 2 illustrates the framework used to assess CMs effectiveness in increasing inclusion in mobility and road space planning. In **Phase 0** a real-world experiment to test a suite of CMs was co-designed in collaboration with key individuals responsible for public engagement, transport and planning including administrators alongside representatives from special interest groups including UN Environment's Share the Road and NGOs. These decision makers worked alongside teams of CM experts to identify suitable physical locations and mobility challenges around which to structure the work using a ranked long list to select two locations where CM interventions would take place based upon the criteria outlined in table S2. To enable reflection on the additionality and unique contribution of CMs to inclusion, governance and outcomes, control sites were similarly identified having comparable mobility issues to the intervention (case) locations. This allowed comparison of CMs to 'business-as-usual' engagement and planning processes more typically undertaken by the relevant authorities in each city.

Table S2

Table S2. Description of the criteria used in co-designing site selection.

Criteria	Description
Context	- Spatial setting<br>- Sites challenges (the mobility issues affecting that location)
Infrastructure interventions benefits	- Status of either proposed or existing improvements designed to improve mobility or safety<br>- Potential benefits of using new methods to inclusively identify innovative solutions and the impact improved co-designed infrastructure might have.

Supplementary Materials S3

Q-Sort Results

The different types of participants who took part in the Q-sort for each time step can be seen in table S2.

Table S3.1: Participant numbers by city for the Q-sort activity

Type of participant	Q-sort 1 Nairobi	Q-sort 2 Nairobi	Q-sort 1 Kampala	Q-sort 2 Kampala	Q-sort 1 Total	Q-sort 2 Total
City planner	6	5	3	3	9	8
Transport planner	7	5	3	3	10	9
NGO	1	0	2	1	3	1

Academic	0	0	1	1	1	1
**Total**	**14**	**10**	**9**	**8**	**23**	**20**

The Q-sort 2 participants were all drawn from those who participated in Q-sort 1 to be able to analyse changes in perspectives over time from the same group. These are described below in table's S3.2 and S3.3.

Table S3.1: the Q-sort statements; whether they are transport or **planning** (in bold) related; their respective scores within each factor; and whether these scores are distinct: Con = consensus across the discourses; F1/F2/F3 = statement is distinctive for that discourse in comparison to the other two; none = no significant distinction in any direction. Sig shows significance level. Significance a (p < .05) of different placement on the Q-sort grid by participants that load on a given factor, to where participants that load on other factors have placed the same statement. Significance b at p < .01.

	**Q-Sort 1**						**Q-Sort 2**					
Statements	Distinguishing Statements	Q1-F1	Q2-F2	Q3-F3	Factor	Significance	Distinguishing Statements	Q2-F1	Q2-F2	Q3-F3	Factor	Significance
**1: Road safety and road space planning should aim to minimise congestion in cities**	**f2**	**1**	**2**	**0**	**2**	**a**	Con	1	0	1		
2: It is not necessary to own a car in a city like Kampala/Nairobi – there are many options to travel around	None	-2	-3	-1			None	-1	-2	-1		
3: Taxis and Boda Bodas clog the roads, add to congestion, and should be restricted	**f2**	**0**	**4**	**0**	**2**	**a**	**f2**	**0**	**4**	**-1**	**2**	**a**
4: We need more and better road space for the car	**f2**	**-3**	**-1**	**-3**	**2**	**a**	**f1**	**-4**	**-1**	**-2**	**1**	**a**
**5: From the planning to implementation stage we should widen engagement through workshops and using the media**	**f2**	**0**	**3**	**1**	**2**	**a**	None	0	1	2		
6: Taxis and Boda Bodas should be given greater priority on our roads	Con	-2	-4	-2			**f2**	**-1**	**-4**	**-1**	**2**	**a**
7: We should build more roads to make driving easier and the roads less congested	**f1**	**-4**	**-2**	**-1**	**1**	**a**	**f2**	**-3**	**-1**	**-4**	**2**	**b**
**8: We should use more creative approaches to engage with a wider range of stakeholders in road space planning**	Con	3	2	3			Con	2	3	3		
**9: Current planning processes focus on engineers with no consultation of non-motorised-transport users**	**f3**	**-1**	**0**	**2**	**3**	**a**	**f1**	**0**	**1**	**3**	**1**	**a**
**10: The most effective engagement approach is formal open days to showcase ongoing and planned road works**	**f1**	**1**	**0**	**-1**	**1**	**a**	**f1**	**-1**	**2**	**1**	**1**	**a**
11: There are no adequate facilities for cycling in the city and cycling on the road is too dangerous	**f1**	**4**	**2**	**1**	**1**	**a**	None	1	3	2		
**12: We need more local level engagement through open community meetings and creative planning approaches**	Con	2	2	1			**f3**	**1**	**1**	**4**	**3**	**b**
**13: Existing engagement approaches for road space planning already allows all stakeholders to contribute**	**f1**	**-2**	**-3**	**-3**	**1**	**a**	**f1**	**-3**	**0**	**0**	**1**	**a**

14: You should be able to walk anywhere you need to go safely using well-planned facilities	**f3**	**2**	**3**	**0**	**3**	**a**	Con	2	2	2		
15: Our current public transport network is not good enough to make car ownership unnecessary	None	3	1	0			**all**	**2**	**0**	**-2**	**4**	**a**
16: City spaces where residents and visitors feel safe to move around prosper both economically and socially	None	3	1	3			Con	3	3	2		
17: Only people who have no other options tend to walk. Walking should be the lowest priority for planning	Con	-4	-4	-2			Con	-3	-2	-3		
**18: Formal consultations for road planning initiatives are often not inclusive and only very few voices are heard**	**f3**	**0**	**1**	**4**	**3**	**a**	None	1	-1	0		
**19: Current road safety and road space planning processes are effective in ensuring all stakeholders get involved**	**f3**	**0**	**-1**	**-3**	**3**	**a**	None	-4	-2	-1		
**20: Road safety and road space planning are important but should be left to experts to decide on the best solutions**	**f2**	**-3**	**0**	**-4**	**2**	**a**	Con	-2	-3	-4		
21: Driving a car gives people greater accessibility in the city, enabling them to live busy lives	None	-1	-1	-2			Con	-2	-3	-2		
22: Being in a car stuck in traffic is better than riding a bus stuck in traffic	Con	-1	-2	-2			Con	-2	-1	-1		
**23: Road safety & space planning should be representative of the % of people using different means of transport**	**f3**	**1**	**1**	**4**	**3**	**b**	**f2**	**3**	**0**	**3**	**2**	**a**
24: Public transport is the only feasible way to get around the city	**all**	**0**	**-3**	**-1**	**4**	**a**	None	-1	0	0		
25: Driving is tiring and stressful – time spent driving is often the worst part of people's day	None	0	0	-1			**f2**	**0**	**-2**	**0**	**2**	**a**
26: Cars are given too much priority on roads	**f3**	**4**	**3**	**2**	**3**	**b**	**f1**	**4**	**1**	**0**	**1**	**a**
27: I would much prefer if I could walk, cycle and use public transport all the time and never travel by car	None	1	0	0			**f1**	**4**	**2**	**1**	**1**	**b**
**28: The best engagement methods for city planning are talking to different groups at informal events**	**f3**	**-1**	**-2**	**1**	**3**	**a**	**all**	**0**	**-3**	**4**	**4**	**a**
29: East African cities typically lack safe and appropriate non-motorised-transport provision	None	2	4	2			**f3**	**2**	**1**	**-1**	**3**	**b**
30: A city where the majority of people feel safe to cycle around is ideal	Con	1	1	0			None	3	0	1		
**31: City planners currently only engage with local government officials and engineers for road planning and construction**	**f3**	**-2**	**-1**	**2**	**3**	**a**	**f2**	**-1**	**-1**	**0**	**2**	**b**
**32: Current planning processes don't include effective engagement. Plans just appear from boardrooms**	**f3**	**-1**	**-2**	**1**	**3**	**a**	**all**	**0**	**2**	**-3**	**4**	**a**
**33: Road safety and road space planning is not very important for city development**	**f2**	**-3**	**-1**	**-4**	**2**	**a**	Con	-2	-4	-3		
**34: We need to widen involvement in road and space planning to take a wider range views into consideration**	**f2**	**2**	**0**	**3**	**2**	**a**	None	1	4	1		

Table S3.3: Qualitative descriptions of the Q-sort perspectives (Q-statement number in [S]) and change from Q1 to Q2.

**Factor 1**	**Factor 2**	**Factor 3**
	**Q-Sort1**	
**Mass Transit Futures**	**Congestion Smasher**	**Inclusive Planning is the Answer**
**Overall Summary**	**Overall Summary**	**Overall Summary**
Sees shortfalls in current engagement around planning and believes NMT should receive greater emphasis in city road space plans – rather than cars.	Wants to widen planning engagement to identify congestion reducing solutions for a mixed mobility future.	Wants planning reforms to improve engagement using mixtures of approaches to get greater inclusion.
Planning Perspective	*Planning Perspective*	*Planning Perspective*
Believes in open inclusive engagement [S10] but thinks existing approaches currently don't allow for this [S13].	Strongly believes in the need to widen engagement opportunities [S5] with the aim of identifying solutions to congestion [S1].	Believes that current planning is not effective at representing all users [S18, 19,23] instead focussing upon engineers solutions and car drivers [S9,31,32] and should become less formal [S28].
Transport Perspective	*Transport Perspective*	*Transport Perspective*
Strongly against car centric developments [S4,7] and believes NMT [S13] and public transport [S24] are the future for urban mobility.	Strongly disagrees that public transport is the only mobility solution [S24] and is strongly against current informal service providers – blaming them for congestion [S3].	Believes the current system favours the car [S26] but does not hold strong views on the ideal solution.
	**Q-Sort2**	
**More Inclusive Planning for a Car Free Future**	**Creative Congestion Smashing**	**Inclusive Creative Planning is the Answer**
**Overall Summary**	**Overall Summary**	**Overall Summary**
Pro-NMT and public transport and anti-car with a belief that current planning approaches are ineffective.	Planning is critical and should be improved with creative engagement. Informal transport should be restricted to promote walking and reduce congestion.	Better community engagement would improve planning and creative approaches could achieve this to improve walking options.
Planning Perspective	*Planning Perspective*	*Planning Perspective*
Existing approaches are ineffective for engagement [S10,13] and more creative approaches are needed [S8].	Planning is critical for city development [S33]. Current non-inclusive [S31,32] engagement is ineffective and could be improved with creativity [8] but full representation is not essential [S23].	Local community engagement is key [S12] with informal events being effective [S28]. Creative methods would widen engagement [S8, S20] making them more representative [S23].
Transport Perspective	*Transport Perspective*	*Transport Perspective*
Cars are given too much priority and roadspace [S4,26]. Strong preference for NMT and public transport solutions [S27, 14,16] to encourage economic and social vibrancy [S16].	Taxis and Boda bodas need to be restricted to reduce congestion [S3,6]. Walking facilities should be improved [S14,17] but driving should be part of the mobility mix [S25].	Public transport makes car ownership unnecessary [S15]. Road building is not the solution [S7] and walking infrastructure should be improved [S14,16,17].

The Q-sort analysis revealed a number of changes that had occurred in our participant's perspectives on transport and planning over the 12 months of the project. Between the two assessment stages beliefs on the transport mix required to improve mobility did not change significantly with two clear camps (a belief in NMT and mass transit and a more mixed mobility future) with a third less prescriptive group. However, on the topic of planning and engagement there was a definite shift from underlying concerns that the business-as-usual approaches were not effective or inclusive to a consensus that creative methods could offer some solutions to these deficits. Table S5 shows a breakdown of the results per city of the changes between the scores for each statement between the 1st and 2nd Q-sort.

Table S3.4: Mean (Mn) value and change (Ch) in individual statement Q-sort scoring (Q1 = Qsort 1; Q2 = Qsort2) per city and total (N = Nairobi; K = Kampala; all = total). Data for statements related to **planning** are in **bold**.

S #	Mn Q1 all	Mn Q2 all	Mn Q1 N	Mn Q2 N	Mn Q1 K	Mn Q2 K	Ch Q1-Q2 all	Ch Q1-Q2 N	Ch Q1-Q2 K
	1.5	1.2	1.6	1.6	1.3	0.8	-0.3	0.0	-0.6
2	-1.4	-1.3	-1.1	-1.3	-1.8	-1.4	0.1	-0.2	0.4
3	0.9	1.0	-0.4	-0.2	2.8	2.5	0.1	0.2	-0.3
4	-2.7	-2.6	-3.0	-3.0	-2.3	-2.0	0.2	0.0	0.3
**5**	**1.4**	**1.3**	**1.2**	**1.5**	**1.7**	**1.1**	**-0.1**	**0.3**	**-0.5**
6	-1.9	-2.0	-1.6	-1.4	-2.3	-2.8	-0.1	0.2	-0.4
7	-2.3	-2.5	-2.3	-3.0	-2.3	-1.9	-0.2	-0.7	0.5
**8**	**1.9**	**1.8**	**1.8**	**1.9**	**2.0**	**1.8**	**0.0**	**0.1**	**-0.3**
**9**	**-0.3**	**0.8**	**0.0**	**0.4**	**-0.7**	**1.4**	**1.1**	**0.4**	**2.0**
**10**	**-0.1**	**0.2**	**-0.3**	**-0.3**	**0.1**	**0.8**	**0.3**	**0.0**	**0.6**
11	2.1	1.8	1.9	1.6	2.3	2.0	-0.3	-0.3	-0.3
**12**	**1.5**	**1.6**	**1.5**	**1.8**	**1.4**	**1.4**	**0.1**	**0.3**	**-0.1**
**13**	**-2.2**	**-1.3**	**-2.1**	**-1.6**	**-2.3**	**-1.0**	**0.8**	**0.5**	**1.3**
14	1.9	2.2	1.9	2.4	1.9	2.0	0.4	0.5	0.1
15	1.6	0.7	1.4	1.2	1.9	0.1	-0.9	-0.2	-1.8
16	2.2	2.5	2.4	2.3	1.9	2.8	0.3	-0.1	0.9
17	-2.9	-2.6	-3.1	-3.1	-2.4	-1.9	0.3	0.0	0.6
**18**	**1.3**	**0.4**	**1.6**	**0.6**	**0.8**	**0.1**	**-0.9**	**-1.0**	**-0.7**
**19**	**-1.5**	**-2.1**	**-1.7**	**-2.3**	**-1.1**	**-1.9**	**-0.6**	**-0.6**	**-0.8**
**20**	**-2.3**	**-2.4**	**-2.4**	**-2.3**	**-2.0**	**-2.5**	**-0.1**	**0.1**	**-0.5**
21	-1.4	-1.8	-1.5	-1.8	-1.2	-1.8	-0.4	-0.3	-0.5
22	-1.1	-1.7	-1.1	-2.3	-1.1	-1.0	-0.6	-1.2	0.1
**23**	**1.5**	**1.6**	**1.9**	**1.9**	**0.9**	**1.1**	**0.1**	**0.0**	**0.2**
24	-1.0	-0.2	-0.7	-0.4	-1.6	0.0	0.8	0.3	1.6
25	-0.3	-0.7	-0.4	0.0	-0.2	-1.5	-0.4	0.4	-1.3
26	2.3	2.1	2.5	2.4	2.1	1.6	-0.3	-0.1	-0.5
27	0.9	1.8	0.9	1.7	1.0	2.0	0.9	0.8	1.0
**28**	**-0.5**	**-0.3**	**-0.1**	**0.9**	**-1.1**	**-1.8**	**0.2**	**1.0**	**-0.6**
29	1.8	1.3	1.5	1.4	2.3	1.3	-0.5	-0.1	-1.1
30	1.5	1.3	1.6	1.8	1.2	0.8	-0.1	0.2	-0.5
**31**	**-0.5**	**-1.0**	**-0.4**	**-0.7**	**-0.7**	**-1.4**	**-0.5**	**-0.3**	**-0.7**
**32**	**-0.6**	**-0.1**	**-0.2**	**-0.5**	**-1.2**	**0.4**	**0.5**	**-0.3**	**1.6**
**33**	**-2.6**	**-2.8**	**-3.1**	**-2.8**	**-1.7**	**-2.8**	**-0.2**	**0.3**	**-1.1**
**34**	**1.3**	**1.8**	**1.9**	**1.9**	**0.4**	**1.6**	**0.4**	**0.0**	**1.2**

Comparing between cities the largest opinion shifts (reflected in the weightings) occurred in Kampala. Taking the absolute value of change (positive modulus value of original real number) indicates the largest changes in opinion were in Kampala where the total change in statement scores is 24.9 compared to Nairobi at 11.0 (note there are less participants in Kampala) with this difference statistically significant $t(55)=4.03$, $p=0.000$).

Comparing between the Q sort weightings only for statements linked to planning (n=15) (bold in table 7) the change in absolute mean value for Nairobi was 5.2 whilst for Kampala it was 12.2. This indicates larger statistically significant ($t(28)=2.93, p=0.003$) shifts in opinions in Kampala than Nairobi.

The participants of the Q-sort activity also described why they most agreed or disagreed with particular statements. For Q-sort 1 in Nairobi, eight participants (57%) placed at least one planning and engagement related statement in their top or bottom ranked statement dropping to six participants (54%) in the repeat survey. In Kampala the figures were eleven participants (91%) in Q-sort 1, increasing to 100% (ten participants) in Q-sort 2. This indicates that planning issues were more pressing for our Ugandan participants.

The concourse of Q-sort statements was developed from sources including: Existing published literature on transport and planning; previous questions utilised in relevant UK and European surveys; and participant quotes from the co-design workshop. Overall thirteen statements represented beliefs on engagement in planning whilst the other 21 statements identified perceptions of transport modal priorities, road safety or urban design goals. The Q-sort was analysed using factor analysis to identify belief groupings. Changes in individual Q-statement weightings between were analysed to identify shifting beliefs.

Supplementary Material S4

Scoring of CM for Inclusion Benefits and Governance Benefits

Individual activities were monitored and evaluated continuously during all phases. Outcomes were assessed internally by the project team (Phases 1 and 3) and evaluated by a wider range of project stakeholders including city planners, transport NGOs, project participants (e.g. teachers from engaged Kampala schools) at Phase 4.

These reflections have been scored by the academic project team members for impacts on widening inclusion using the metrics connected to our evaluation framework, specifically: The types and number of outputs generated (e.g. number of maps, variety of participants) (linked to (c)); engagement outcomes (linked to (b), (d) and (e)); levels of participation (number of participants; or numbers of social media messages etc.) (linked to (a) and (d)). This analysis was supported by feedback recorded in impact stories, videos, social and print media from events or content received from stakeholders including local users of the case study sites.

The scores for individual methods can be seen below in table S4.1 and S4.2 with summary statistics for method categories in table S4.3 and S4.4.

Table S4.1: Individual creative methods scored for impacts on individual dimensions of Hammond et al. framework by the academic partners in the project team.

Score of two indicates a primary outcome or impact; a secondary or peripheral impact scores one; no impact or benefit scores zero.

				(a) engaging participants in relevant activities;	(b) cultivating relationships of mutual trust, respect, and power;	(c) creating new (forms of) knowledge;	(d) building individual or community capacities	(e) initiating community action and change.
1	Digital	2-way	Mine craft model	2	1	2	1	
2	Digital	2-way	Urban dialogues	2	2	1	1	1
3	Digital	2-way	Social media content	2		1	1	
4	Digital	2-way	Digital story collection	2	2	2	1	
5	Digital	2-way	Wearable cameras	2	2	2	1	
6	Digital	1-way	Time-lapse videos			2		
7	Digital	1-way	Drone imagery			2		
8	Digital	1-way	VR streetscapes			2		
9	Digital	1-way	Photo hangout outputs	1		2	1	1
10	Digital	1-way	Digital story outputs	1	1	2		1
11	Digital	1-way	Infographics			2	1	2
12	Digital	1-way	Street designs	1	2			2
14	Physical	Objects	On-street architectural models	2	2	2		
15	Physical	Objects	PGIS maps	2	1	2		1
16	Physical	Objects	Urban guerrilla signage				1	2
17	Physical	Objects	Street art canvases	2		1	1	1
18	Physical	Objects	Pop up feedback displays	1	1	1	1	1
19	Physical	Objects	Photo hangouts	1		2		
20	Physical	Objects	Architectural designs					1
21	Physical	Objects	Comics & cartoons	2			2	2
22	Physical	Objects	Infographics				1	2
23	Physical	Activities	Placemaking: Streetscape testing	2	2	1	2	2
24	Physical	Activities	3D-Zebra Crossing				1	2
25	Physical	Activities	Urban dialogues	2	2	1	1	1
26	Physical	Activities	Theatre	2	2	1	1	1
27	Physical	Activities	Creative play	2	1	1	2	2
28	Physical	Activities	Design competition		1		1	2
			Primary Purpose	13	8	12	3	9
			Secondary Purpose	5	6	8	15	9

Table S4.2: Individual creative methods scored by impact on individual dimensions of Fung's framework.

	D = Digital; P = Physical	1 = 1 way; 2 = 2-way; O = Object; A = Activity	**Method**	**Participant Selection (Level of Inclusion)**	*1= State;*	*2 = Professional & Lay stakeholders*	*3 = Random selection to open self-selection*	*4 = Diffuse publics*	**Communication & Decision modes (Intensity of participation)**	*1= Listen as spectator;*	*2 = Express or develop preferences;*	*3= Aggregate, bargain; Deliberate & negotiate;*		**Extent of authority & power (Level of authority)**	*1 = Personal benefit*	*2 = Communicative influence; Advise & consult;*	*3 = Co-governance & Direct authority*
1	D	2	Mine craft model			2					2				1		
2	D	2	Urban dialogues			2					2					2	
3	D	2	Social media content					4		1						2	
4	D	2	Digital story collection			2					2				1		
5	D	2	Wearable cameras				3			1						2	
6	D	1	Time-lapse videos			2				1						2	
7	D	1	Drone imagery			2				1						2	
8	D	1	VR streetscapes			2				1						2	
9	D	1	Photo hangout outputs					4		1						2	
10	D	1	Digital story outputs					4		1						2	
11	D	1	Infographics					4			2				1		
12	D	1	Street designs				3				2					2	
13	P	O	On-street architectural models				3				2					2	
14	P	O	PGIS maps				3				2				1		
15	P	O	Urban guerrilla signage					4		1					1		
16	P	O	Street art canvases				3			1					1		
17	P	O	Pop up feedback displays					4		1					1		
18	P	O	Photo hangouts			2				1					1		
19	P	O	Architectural designs			2						3				2	
20	P	O	Comics & cartoons					4		1					1		
21	P	O	Infographics					4		1					1		
22	P	A	Placemaking					4			2					2	
23	P	A	3D-Zebra Crossing					4								2	
24	P	A	Urban dialogues				3					3				2	
25	P	A	Theatre				3			1					1		
26	P	A	Creative play				3			1					1		
27	P	A	Design competition			2						3				2	

Table S4.3: Grouped creative methods total scores by impact on individual dimensions of Hammond et al. framework as totals and percentages of maximum score. This percentage data forms the basis of figure 5a. in the main document.

		(a) Participant relevance	(b) Relationships cultivation	(c) Knowledge creation	(d) Capacity building	(e) Community action	Max. Potential Score	(a) Participant relevance%	(b) Relationships cultivation%	(c) Knowledge creation%	(d) Capacity building%	(e) Community action%
Digital	2-way	10	7	8	5	1	10	100%	70%	80%	50%	10%
Digital	1-way	3	3	12	2	8	16	19%	19%	75%	13%	50%
Physical	Objects	8	4	8	6	12	18	44%	22%	44%	33%	67%
Physical	Activities	8	8	4	8	10	12	67%	67%	33%	67%	83%

Table S4.4: Grouped creative methods total median scores across 3 dimensions of Fung's framework.

		**Median Score - Participant Selection**	**Median Score - Communication & Decision modes**	**Median Score - Extent of authority & power**
Digital	2-way	2	2	2
Digital	1-way	3.5	1	2
Physical	Objects	3	1	1
Physical	Activities	3	2	2
